# Sunflower Oil but Not Fish Oil Resembles Positive Effects of Virgin Olive Oil on Aged Pancreas after Life-Long Coenzyme Q Addition

**DOI:** 10.3390/ijms161023425

**Published:** 2015-09-29

**Authors:** Adrián González-Alonso, César L. Ramírez-Tortosa, Alfonso Varela-López, Enrique Roche, María I. Arribas, M. Carmen Ramírez-Tortosa, Francesca Giampieri, Julio J. Ochoa, José L. Quiles

**Affiliations:** 1 Department of Physiology, Institute of Nutrition and Food Technology “Jose Mataix”, University of Granada, Biomedical Research Center, lab. 250, Avda. Conocimiento s/n, 18100 Armilla, Granada, Spain; E-Mails: kulturadrian@hotmail.com (A.G.-A.); alvarela@ugr.es (A.V.-L.); jjoh@ugr.es (J.J.O.); 2 Department of Pathology, Complejo Hospitalario de Jaén, 23007 Jaén, Spain; E-Mail: cesarl.ramirez.sspa@juntadeandalucia.es; 3 Bioengineering Institute, University Miguel Hernandez and CIBEROBN (CB 12/03/30038, Fisiopatología de la Obesidad y la Nutrición, Instituto de Salud Carlos III), 03202 Elche, Spain; E-Mails: eroche@umh.es (E.R.); miarribas@umh.es (M.I.A.); 4 Department of Biochemistry and Molecular Biology II, Institute of Nutrition and Food Technology “Jose Mataix”, University of Granada, 18071 Granada, Spain; E-Mail: mramirez@ugr.es; 5 Dip. Scienze Cliniche Specialistiche ed Odontostomatologiche, Università Politecnica delle Marche, 60121 Ancona, Italy; E-Mail: f.giampieri@univpm.it

**Keywords:** β-cell, endocrine pancreas, exocrine pancreas, inflammation, pancreatic aging, pancreatic fibrosis of the elderly, fat, dietary lipids

## Abstract

An adequate pancreatic structure is necessary for optimal organ function. Structural changes are critical in the development of age-related pancreatic disorders. In this context, it has been reported that different pancreatic compartments from rats were affected according to the fat composition consumed. Since there is a close relationship between mitochondria, oxidative stress and aging, an experimental approach has been developed to gain more insight into this process in the pancreas. A low dosage of coenzyme Q was administered life-long in rats in order to try to prevent pancreatic aging-related alterations associated to some dietary fat sources. According to that, three groups of rats were fed normocaloric diets containing Coenzyme Q (CoQ) for two years, where virgin olive, sunflower, or fish oil was included as unique fat source. Pancreatic samples for microscopy and blood samples were collected at the moment of euthanasia. The main finding is that CoQ supplementation gives different results according to fat used in diet. When sunflower oil was the main fat in the diet, CoQ supplementation seems to improve endocrine pancreas structure and in particular β-cell mass resembling positive effects of virgin olive oil. Conversely, CoQ intake does not seem to improve the structural alterations of exocrine compartment previously observed in fish oil fed rats. Therefore CoQ may improve pancreatic alterations associated to the chronic intake of some dietary fat sources.

## 1. Introduction

The pancreas is a key organ in nutrient digestion as well as in nutrient utilization. This dual function can be performed due to the presence of two compartments: the exocrine and the endocrine pancreas. The exocrine portion is the most abundant (98% of pancreatic mass) and is formed by interstitial mesenchymal, ductal and acinar cells, these last producing enzymes responsible for food digestion in the duodenum. The endocrine compartment is formed by cell aggregates (also called islets of Langerhans) disseminated through the exocrine structure and formed by different cell types specialized in the biosynthesis and secretion of specific hormones: insulin by β-cells (around 60% of islet mass), glucagon by α-cells (30% of islet mass), somatostatin by δ-cells, pancreatic polypeptide by γ-cells and ghrelin by ε-cells, these last only appearing during embryogenesis [1,2]. During the life cycle, persistent bad habits in diet and life style can predispose to the appearance of certain pathologies that can disturb independently both pancreatic compartments and accelerate the organ aging process. In addition, it is obvious that due to the close proximity, pathological states affecting one of the pancreatic compartments will inevitably affect the other [3].

In this context, dietary habits have shown to exert effects on both compartments. On one hand, exocrine pancreatic compartment have been shown to be disturbed by alcohol abuse [4] that lead to destruction of functional exocrine pancreatic tissue resulting in nutrient malabsorption in the intestine [5]. On the other hand, endocrine pancreatic compartments, particularly the β-cells, are disturbed in type 2 diabetes mellitus [6], which is closely related to an excessive and unbalanced intake of high calorie nutrients such as fat and sugars [7]. Concerning fat in the diet, depending of the nature of this nutrient, different effects have been reported both on the endocrine as well as on the exocrine function. In this sense, different intervention and epidemiological studies clearly state that monounsaturated fat-rich diets improve insulin sensitivity [8,9,10,11,12] and lead to lower glycaemia and insulin requirements in type 2 diabetic subjects [13,14]. On the other hand, as far as exocrine pancreas compartment is concerned, extensive work has been carried out mainly comparing virgin olive oil against sunflower oil from the point of view of exocrine pancreatic secretion [15]. Authors demonstrated that exocrine pancreatic secretory activity in response to food was greater in dogs fed on a diet rich in sunflower oil than in animals given the same diet with virgin olive oil [16,17]. These authors, working on rats found that chronic intake of diets differing only in the type of fat (olive oil or sunflower oil) influences the fatty acid profile of pancreatic cell membranes; with rats fed the olive oil diet showing higher levels of oleic acid, whereas those fed sunflower oil had increased linoleic acid and PUFA n-6 contents. In these animals, exocrine pancreatic secretion in anesthetized animals was also affected [18]. Regarding dietary fat type in the aged pancreas, we have previously reported the effects of life-long feeding rats with virgin olive, sunflower or fish oil on the endocrine function and histology of the pancreas [19]. The main finding was related to the beneficial effect of monounsaturated fat-rich diets in maintaining pancreatic microstructure and endocrine function. Thus, animals fed on sunflower oil showed higher β-cell numbers and insulin content compared with those fed on virgin olive oil. In addition, animals fed on fish oil developed acinar fibrosis and presented macrophage infiltrates in peri-insular regions, also compared with animals fed on virgin olive oil.

Coenzyme Q (CoQ) is the general term that refers to a class of benzoquinones widely distributed in the phospholipidic bilayers of cellular and organelle membranes of living organisms [20,21,22]. CoQ is composed of a redox active quinoid moiety linked to an isoprenoid side chain (2,3-dimethoxy-5 methyl-6-multiprenyl-1-4-benzoquinone) comprising six to ten units, depending on the species [21,23]. In humans, the predominant form of CoQ comprises 10 isoprenoid repeats in the side chain [24] and it is also referred as ubiquinone, meanwhile in rats the predominant form contains nine isoprenoid subunits [25]. CoQ is synthesized by the mevalonate pathway and presents a varying distribution among body tissues [22,26]. CoQ plays an instrumental role in mitochondrial energy production by transferring electrons from respiratory complexes I and II to complex III of the electron transport chain with simultaneous proton translocation to the intermembrane space, contributing to the generation of the transmembrane proton gradient that drives the synthesis of ATP from APD phosphorylation [27]. Ubiquinol is the two-electron reduction product of CoQ [28], functioning as an antioxidant in mitochondria and lipidic membranes [29] and representing the 80%–90% of the CoQ pool in many fluids and tissues including plasma, liver, pancreas and intestine [30,31]. However, the partially reduced form of CoQ is the primary source of superoxide radical in the mitochondrial respiratory chain [32]. In this context, the oxidized form of CoQ predominates in brain and lung [33]. Several reports have implicated direct or indirectly CoQ deficiency in the development of pathologies and physiological states associated with an impaired mitochondrial bioenergetic function such as aging and age-associated diseases [26,34,35,36,37,38]. Concerning aging, the most extended hypothesis is that mitochondrial respiration tend to decline during aging as well as mitochondrial CoQ levels, resulting in a decrease of ATP biosynthesis and an increase in superoxide generation with associated damage in biological macromolecules such as membrane lipids, proteins and DNA [39,40]. In this context, results from several studies indicate that CoQ intake results in an amelioration of age-related disorders [41,42,43,44]. In the same way, it has been previously reported how CoQ feeding may counteract some alterations associated to age in different tissues in the rat, like the liver [45], brain [46], heart [47,48], skeletal muscle [49], and periodontal tissue [50] after feeding pro-oxidant fats like sunflower oil (including an impaired function and an increased oxidative stress at the mitochondria); or even expanding lifespan [51].

According to the above-mentioned variations in pancreas structure and function after lifelong feeding on different dietary fats, and the potentially beneficial effects on these aspects by CoQ supplementation, the present study was designed with the aim to compare three groups of rats maintained for two years with isocaloric diets containing as the only dietary fat virgin olive (VOO), sunflower (SO) or fish oils (FO), all of them supplemented with CoQ.

## 2. Results and Discussion

### 2.1. Body Weight Evolution and Adaptation to the Diet

After the 24 month follow-up period, rats fed on FO + CoQ displayed significantly higher body weight (624 ± 20 g) than those receiving VOO + CoQ (496 ± 20 g) and SO + CoQ (499.0 ± 6.6 g). However, from the observation of food spillage, no differences concerning food intake were inferred between dietary groups or in relation to age.

As it has been shown, dietary fat sources as those used in this study are rich in different groups of fatty acids [18,52]. Because dietary fat composition was the main differential parameter in all groups of animals, we needed to verify a proper adaptation to the diet of the animals in order to attribute the possible differences among groups to them. For this reason, circulating fatty acids profile was analyzed. Results showed that for C18:1n-9 (oleic acid, the most representative fatty acid found in virgin olive oil), the highest percentage was found for VOO fed animals (13.3% ± 2.8%), that was significantly higher than that found in SO (4.9% ± 1.1%) and FO (6.1% ± 1.7%) animals. Concerning C18:2n-6 (linolenic acid, the most representative fatty acid found in sunflower oil), SO fed animals showed significantly higher percentage (14.6% ± 0.6%) than VOO (8.4% ± 1.9%) and FO (2.6% ± 0.5%) groups. Finally, for C22:6n-3 (docosahexaenoic acid, mostly present in marine species, FO group led to the significantly highest percentage (8.1% ± 0.5%), compared with VOO (1.8% ± 0.4%) and SO groups (0.3% ± 0.1%). Something similar was found for the other typical marker for fish oil, namely, eicosapentaenoic acid (EPA); the FO group reported the highest percentage (9.9% ± 0.7%), compared with VOO (0.2% ± 0.1%) and SO groups (not detected). This lipid profile resembles that present in the original composition of oils used in the diets and it would indicate a proper adaptation of the rats to the different dietary fats as previous studies [18,53,54].

### 2.2. Circulating Hormone Levels and Biochemical Parameters

A major pancreatic alteration associated to aging is type 2 diabetes melllitus that appears when adaptation of the β-cell mass fails to compensate the increased insulin demand, resulting in β-cell apoptosis [6]. Increased insulin circulating levels and hyperleptinemia along with other parameters have been described in obesity, glucose intolerance, insulin resistance, disruption of the adipoinsular axis or prediabetes [55]. According to that, insulin resistance and hyperinsulinemia are components of the age-related metabolic disorders such as glucose intolerance, dyslipidemia, obesity, cardiovascular disease, and metabolic syndrome [3,56,57,58,59] that present a high risk of evolving towards type 2 diabetes [19]. To evaluate the health of the animals concerning these potential risks, both, circulating levels of insulin and leptin, as well as biochemical parameters related to their actions were measured.

Regarding hormone levels, no significant differences were observed in the amounts of circulating leptin found between CoQ supplemented rats fed on VOO and SO or FO, meanwhile supplemented FO-fed animals presented lower circulating insulin values compared with VOO fed rats (Table 1). When the levels of these hormones were studied in animals fed on similar dietary fats without CoQ supplementation [19], it was noted that significantly higher values were found only for leptin in animals fed on SO. Eicosapentaenoic acid (EPA), one of the n-3 polyunsaturated fatty acids, has been shown to stimulate leptin mRNA expression and secretion in 3T3-L1 cells [60]. According to that, it is possible that CoQ increased leptin levels only in animals fed on FO and SO, although no additive effects might be accounted for those fed on FO.

In addition, at the moment of euthanasia, all groups of CoQ supplemented animals displayed very similar glycemia and serum lipids, including triglycerides, cholesterol and phospholipids, but not total lipids, which were lower in FO + CoQ rats than in VOO + CoQ animals (Table 1). Likewise, the calculated HOMA (homeostasis model assessment) index showed significantly lower values in FO + CoQ fed rats (Table 1), which is in accordance with the lower insulin levels. However no clear differences were observed between animal fed on similar dietary fats in absence of CoQ supplementation for the last seven parameters [19]. Thus, from the point of view of circulating hormones and biochemical parameters, it is difficult to establish the role of CoQ supplementation in the three studied dietary fats; in any case, neither group of rats showed a significant pathological situation of insulin resistance or loss of insulin sensitivity [61]. On the other hand, CoQ supplementation seemed to reduce leptin levels associated to a SO-based diet. Leptin is produced and released primarily by adipocytes whose circulating levels are directly proportional to total fat mass [62], but also to the percentage of body fat and BMI [63,64]. Nevertheless, with the present methodological approach, it is not possible to establish a clear explanation for CoQ effects on circulating leptin levels when rats are maintained on a SO-based diet.

**Table 1:** Circulating parameters of rats fed on CoQ supplemented diet based on virgin olive oil (VOO), sunflower oil (SO) and fish oil (FO) at the end of the experimental procedure.

Parameter	VOO	SO	FO
Insulin (pg/mL)	302.7 ± 84.6	212.3 ± 58.7	170.7 ± 47.9 *
Leptin (pg/mL)	14,211.4 ± 2966.2	13,462.9 ± 2275.2	21,580.5 ± 4152.9
Glucose (mM)	6.8 ± 0.5	6.4 ± 0.6	6.4 ± 0.5
HOMA	2.3 ± 0.7	1.6 ± 0.5	1.2 ± 0.4 *
Triglycerides (mM)	1.5 ± 0.3	1.4 ± 0.3	1.1 ±0.1
Cholesterol (mM)	2.2 ± 0.4	2.5 ± 0.1	1.6 ± 0.1
Phospholipids (mM)	1.1 ± 0.1	1.0 ± 0.1	0.8 ± 0.1
Total lipids (mg/dL)	387.1 ± 61.5	438.0 ± 56.4	252.3 ± 33.7 *

Data are presented as mean ± standard error of mean. Abbreviations: HOMA: Homeostatic model assessment; Symbols: * *p* <0.05 respect to VOO fed rats.

### 2.3. Histological Study

#### 2.3.1. Pancreatic Parenchyma

**Figure 1:**
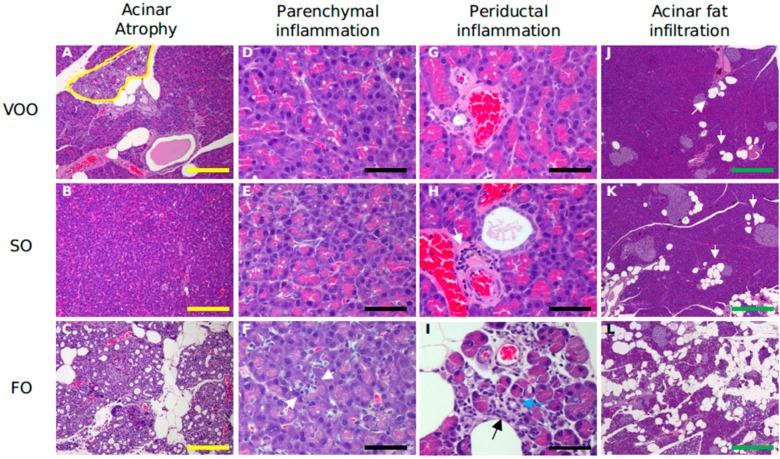
Histological sections of pancreas from rats fed on different dietary fat sources supplemented with Coenzyme Q: virgin olive (VOO), sunflower (SO) and fish oil (FO) after hematoxilin and eosin staining for assessment of acinar atrophy, parenchymal and periductal inflammation and acinar fat infiltration, respectively. (**A**) Minimal acinar atrophy (indicated by a yellow circle); (**B**) Normal pancreatic tissues; (**C**) Severe acinar atrophy, grade 4; (**D**,**E**) Absence of inflammation; (**F**) Inflammatory cells (its presence is indicated by white arrows); (**G**) Absence of inflammation; (**H**) Minimal inflammation with some inflammatory cells (indicated by a white arrow); (**I**) More severe infiltrate (indicated by a black arrow) around the duct (indicated by a blue arrow); (**J**,**K**) Very low number of fatty cells and aggregates (grade 1–2) (indicated by white arrows); (**L**) Severe fat deposits (grade 3–4). Yellow bar = 200 μm; Black bar = 50 μm; Green bar = 500 μm.

**Figure 2:**
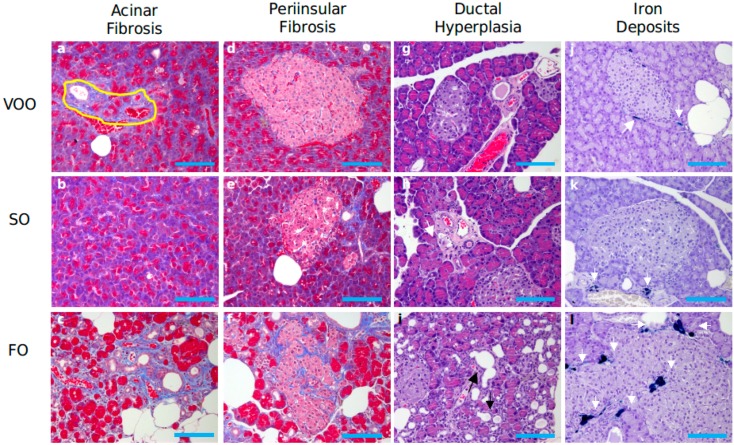
Histological sections of pancreas from rats fed on different dietary fat sources supplemented with coenzyme Q: virgin olive (VOO), sunflower (SO) and fish oil (FO) after Trichrome Masson (**a**–**f**), Hematoxilin and eosin (**g**–**i**) and Perls staining (**j**–**l**) for assessment of acinar and peri-insular fibrosis, ductal hyperplasia and iron deposits, respectively. (**a**) Minimal acinar fibrosis (indicated by a yellow circle); (**b**) Normal pancreatic acini; (**c**) Acinar fibrosis grade 3; (**d**,**e**) Absence of peri-insular fibrosis; (**f**) Moderate fibrosis; (**g**,**h**) Absence of ductular hyperplasia; (**i**) Ductular hyperplasia (indicated by black arrows); (**j**,**k**) Very low number of macrophages stained for hemosiderin (grade 1) with peri-insular distribution (indicated by white arrows); (**l**) High number of macrophages stained for hemosiderin (grade 3–4), (hemosiderin deposited are indicated by white arrows). Blue bar = 100 μm.

**Figure 3:**
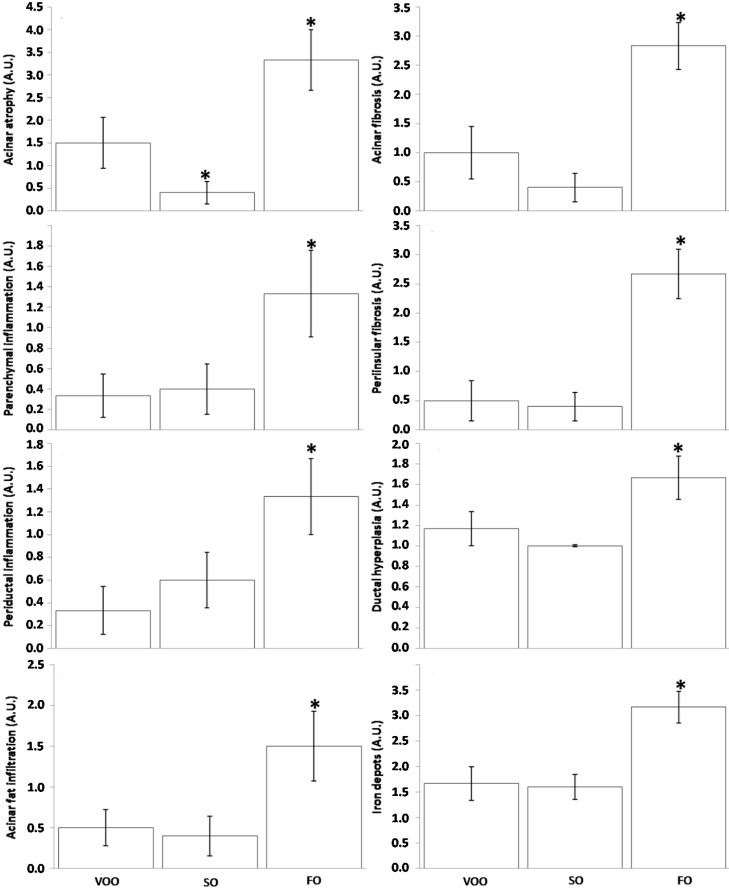
Data from histological evaluations of pancreas from rats fed on different dietary fat sources supplemented with Coenzyme Q: virgin olive (VOO), sunflower (SO) and fish oil (FO). Data are presented as mean ± SEM. A.U. = arbitrary units. * *p* < 0.05 in respect to VOO fed rats.

Histological assessment of pancreas revealed a clear acinar atrophy (Figure 1C) in rats fed on FO-based diet supplemented with CoQ, accompanied by signs of inflammation (Figure 1F,I) and fibrosis, which was affecting the acinar (Figure 2c) but also peri-insular compartment (Figure 2f). In addition, it was also noted an evident degree of hyperplasia in the ductal region (Figure 2i) as well as certain degree of acinar fat infiltration (Figure 1L). In turn, the other two experimental groups generally showed lower alteration degree and no significant differences existed between them for most of the features analyzed. (Figure 3). In this sense, light signs of fibrosis in animals fed on VOO (Figure 2a) have been observed. Additionally, acini scarcely display fat depots (Figure 1J,K) and the ductal tissue and pancreatic blood vessels did not present signs of metaplasia or hyperplasia (Figure 2g,h). The only difference was that a slight degree of acinar atrophy was noted in rats fed with VOO and CoQ (Figure 1A) whereas in those fed with SO and CoQ, there were many samples in which it was absent (Figure 1B). Previously, in rats fed on FO and VOO-based diets, but without CoQ, similar alterations were found [19]. However, SO fed rats did not show significant differences in respect to any of the other group for many of the analyzed features, even they showed a similar degree of ductal hyperplasia that those fed on FO. Consequently, pancreatic alteration degree in this group was intermediate between the other two. Histological alterations observed in FO fed animals, supplemented or not, resemble those observed in pancreatic fibrosis of the elderly [65]. The higher degree of inflammation and fibrosis in pancreas found in animals fed on FO seems contradictory with those from animal studies where n-3 fatty acid-rich diets shows anti-inflammatory effects, although pancreas was not directly studied [66,67,68,69]. However, other studies have also indicated an absence of such effects [70] or even pro-oxidant and pro-inflammatory effects for these types of fatty acids [71,72]; in addition, it has also been observed that some treatment based on them can decrease lifespan [73,74,75,76]. So, the amount, the animal model, age or treatment time seem to be important factors affecting n-3 fatty acid effects that need to be explored in more detail.

Conversely, long-term consumption of diets only based on VOO and SO but supplemented with CoQ, would delay exocrine pancreatic aging in the present model. Pancreatic fibrosis is a complex process in which pancreatic stellate cells (PSCs) play a pivotal role [77,78]. In the normal pancreas, PSCs present a quiescent stage featured by vitamin A-containing lipid droplets, but they can transform into myofibroblast-like cells expressing α-smooth muscle actin [79,80], which are responsible for extracellular matrix deposition and therefore fibrosis [79]. This occurs in response to cytokines and growth factors released by infiltrated inflammatory cells (mainly macrophages) and pancreatic resident cells against cell or tissue damage that stimulate PSCs growth and production of extracellular matrix proteins [81,82,83,84,85,86,87,88]. In turn, activated PSCs are able to produce many inflammatory mediators when they are stimulated by interleukin (IL)-1β and Tumor Necrosis Factor-α (TNF-α), which contributes to accelerating the process of inflammation [89]. In this sense, there is considerable evidence indicating that oxidative stress is implicated in PSC activation [65,66,90,91,92,93,94].

Different antioxidants therapies have been tested demonstrating decreased PSCs and proinflammatory factors both, *in vitro* [90,94] and *in vivo* [87,88,89,90,91,92,93,94,95]. Likewise, histological findings from the present study suggest that long-term addition of a low-dosage of CoQ to a diet with SO as unique dietary fat results in improvement of certain aging indicators in the exocrine compartment of pancreas compared with their corresponding non-supplemented counterparts [19]. Nevertheless, in animals fed on FO and VOO, CoQ supplementation seems to have no effect over pancreatic parenchyma. On the one hand, it could be hypothesized that the FO effect on the exocrine compartment of pancreas was due to a mechanism not related to oxidative stress, or at least that this was not exclusive. On the other hand, it is possible that CoQ does not act as a specific antioxidant for this particular process and/or not have enough to counteract FO effects. Regarding VOO, it might be so beneficial for this pancreatic compartment that CoQ supplements hardly have additional effects, but curiously the degree of acinar atrophy was lower in SO fed rat supplemented with CoQ which suggests that this combination could be even more beneficial. In any case, more studies are needed in order to understand mechanisms underlying differences among dietary fats and CoQ effects.

#### 2.3.2. Immunohistochemical Assessment of Pancreatic Islets

**Figure 4:**
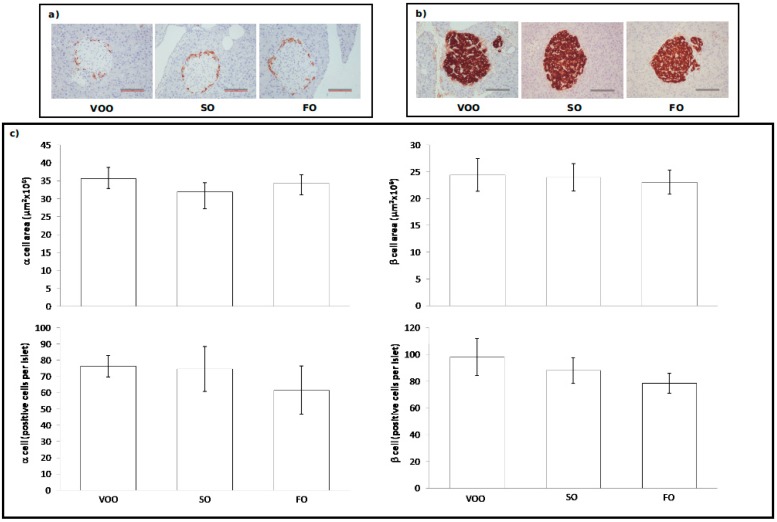
Immunohistochemical assessment in the pancreas of rats fed on different dietary fat sources supplemented with Coenzyme Q: virgin olive oil (VOO), sunflower oil (SO) and fish oil (FO). (**a**) Histological sections with pancreatic isles immunostained for glucagon from rats fed on VOO, SO and FO; (**b**) Histological sections with pancreatic isles immunostained for insulin from rats fed on VOO, SO and FO; (**c**) Quantitative data from histological evaluations of α- and β-cell positive density and area. Data are presented as mean ± SEM. Scare bar = 100 μm.

Concerning pancreatic islets, cell type distribution in islets and their density were assessed by using insulin and glucagon immunohistochemistry to identify β- (Figure 4b) and α-cells (Figure 4a), respectively. In relation to these, no significant differences were found among dietary groups for any of the studied parameters (Figure 4c). This absence of differences is interesting since in the previous study with non CoQ supplemented diets based on similar fats, rats fed on SO presented higher values than those fed on VOO for the β-cell area, as well as a higher number of cells per islet [19]. Age-related disorders of pancreatic endocrine function usually start with a prediabetic state of insulin resistance in which the adaptive response of pancreatic β-cells results in the presence of high levels of circulating insulin (hyperinsulinemia) in order to maintain normoglycemia. Hyperinsulinemia can be achieved by combining different mechanisms that imply expanding β-cell mass by hyperplasia and hypertrophy, enhancing insulin biosynthesis and increasing insulin secretion in response to sustained extracellular nutrient demands [6,96,97,98,99]. However, in SO fed rats from the present study there are no signs of β-cell hypertrophy, nor hyperplasia at all. This finding suggests that CoQ addition to diet prevented age-related β-cell alterations associated to long-life consumption of a SO-rich diet under our experimental conditions. Leptin has been shown to have a proliferative effect on β-cell cultures under certain conditions [100,101,102,103,104]. As mentioned before, it is not possible to establish an explanation for CoQ effect on leptin levels, but this effect on β-cells might derive from it, since this group displayed lower levels than in the non-supplemented counterparts [19].

### 2.4. Pancreatic Contents of Insulin and Glucagon

In order to gain more insight into about how histological changes could affect the endocrine pancreas compartment, main hormones produced by this micro-organ, *i.e.*, insulin and glucagon, were measured. The three groups of animals presented similar pancreatic content for both insulin and glucagon (Table 2). In particular, FO fed animals supplemented with CoQ, that started to develop certain islet affectation, did not show lower hormone content. This suggests that the alterations observed at the level of the peri-insular region have not lead yet to endocrine dysfunction. In addition, and compared to previous determinations in non-supplemented animals from previous studies [19], it was observed that total insulin content in rats fed on VOO and FO supplemented with CoQ was double that in the non-supplemented counterparts. Meanwhile, supplemented and non-supplemented SO fed rats presented similar insulin contents. However in the second case, they were associated with a higher β-cell mass [19]. It has been hypothesized that β-cell hypertrophy could be an adaptive response to the aging situation in an attempt to maintain correct insulin levels in an organ with diminished replicative capacities [103] which would explain observations from the previous study [19]. In turn, here, histological measures related to β-cells were similar among groups and similar to those found in supplemented animals fed FO or VOO [19]. This suggest that CoQ may contribute to specifically increase the content of insulin regardless dietary fat type, but without affecting glucagon that maintained similar levels among dietary groups in both studies. HOMA values, insulin circulating levels and glycaemia were similar to those found in absence of CoQ [19], which suggests that there is no decrease in insulin secretion levels or demands by other tissues. Another possibility is that CoQ improves β-cells capacity to restore insulin reserves. However, because of our experimental design focused on pancreas, it was not possible to elucidate it.

**Table 2:** Total insulin and glucagon pancreatic contents in rats fed on CoQ supplemented diet based on virgin olive oil (VOO), sunflower oil (SO) and fish oil (FO) at the end of the experimental procedure.

Parameter	VOO	SO	FO
Insulin (pg/μg protein)	18,902.5 ± 6989.5	14,144 ± 4588.5	19,576 ± 3720.3
Glucagon (pg/μg protein)	151.8 ± 12.8	143 ± 37.2	129.6 ± 23.8

## 3. Experimental Section

### 3.1. Animals and Diet

Eighteen male Wistar *rats* (Rattus *norvegicus*) initially weighing 80–90 g were housed during 2 years and fed with the semisynthetic and isocaloric AIN-93G (the first two months) and AIN-93M diets [105]. Components of AIN-93G diet in weight percentage were: 38.7% starch, 20% caseine, 13.2% dextrose, 10% sucrose, 8% oil, 5% cellulose (fibre), 3.5% mineral mix, 1% vitamin mix, 0.3% l-cysteine and 0.25% choline bitartrate. This corresponded to a caloric distribution of 43.3% polysaccharides, 11.1% disaccharides, 14.6% monosaccharides, 22.2% proteins and 8.8% lipids. AIN-93M diet presents similar weight composition than AIN-93G except for: 46.6% starch, 14% caseine, 15.5% dextrose, 4% oil and 0.18% l-cysteine. The corresponding caloric composition for AIN-93GM diet was: 51.8% polysaccharides, 11.1% disaccharides, 16.7% monosaccharides, 25.8% proteins and 4.4% lipids.

Three groups of 6 animals each were fed with the described diets presenting different lipid composition: VOO, SO and FO. FO diet was prepared by using pure fish oil ROPUFA 30 (DSM, Kaiseraugst, Switzerland). Sunflower oil was acquired from a local supermarket. Extra virgin olive oil was a kind gift from the agricultural research center “Venta del Llano” from Mengibar, Jaén, Spain. Additionally all diets were supplemented with 50 mg/kg/day of CoQ. CoQ dosage has been chosen according with human intake recommendation for this molecule. Previous studies from our laboratory support the use of this dosage in an aging model in the rat [47,50,52,106]. Animals were placed in a climate-controlled room (20 °C, 12 h dark/12 h light cycle) for two years in collective cages, in groups of three animals per cage, with free access to water. Diet was delivered *ad libitum* for the first two months and then at 25 g/rat/day for the rest of the experiment (in order to avoid overweight). Food intake was indirectly monitored through the weekly body weight control and daily spillage monitoring.

The animals were treated in accordance with the guidelines of the Spanish Society for Laboratory. Animals and the experiment was approved by the Ethical Committee of the University of Granada. The rats were killed by cervical dislocation followed by decapitation, at the same time of the day to avoid any circadian fluctuation. At the end of the experiment, rats underwent overnight fasts and were euthanasized next day. Different tissues and samples were quickly removed for different studies. In our particular case, pancreas was quickly dissected and excised in 3 portions. One portion was snap frozen in liquid nitrogen for protein, insulin and glucagon determinations. The other portions were fixed in formaldehyde for immuno-histological analyses. Blood was collected in EDTA-coated tubes and the plasma was centrifuged at 1750× *g* for 10 min. Plasma samples were stored at −80 °C until analyzed.

### 3.2. Histological Assessments

Pancreatic tissue taken from each rat was fixed in 10% formalin and embedded in paraffin to cut several 4 μm sections. For histological examinations, the sections were stained with haematoxylin and eosin, Masson’s Trichrome and Perls staining. Evaluation of the pathological changes was performed by two experienced pathologists who were blinded to the treatment groups. When there were discrepancies, they discussed among themselves and reached a consensus outcome. Total fields per section (the full slide) were observed and the entire available pancreas was studied in order to avoid a focal observation of the same. Acinar atrophy was scored as 0, absent; 1, cytoplasmic basophilia reduction, acinar size reduction and reduction in the content of acinar zymogen granules; 2, acinar vacuolization, early destruction of acini, inflammation and early onset of fibrosis; 3, moderate; 4, severe with metaplasia and/or acinar dilatation (transformation of acini in cystic/ductular structures coated with cuboidal or flattened cells or the formation of small glands lined by cuboidal cells in a microcystic pattern or “honeycomb”). Parenchymal and periductal inflammation: number of groups of inflammatory cells into parenchyma or around of ducts in total fields per section. Acinar fat infiltration (presence of adipocytes in the pancreatic parenchyma) was scored as 0, absent; 1, very low number of cells and aggregates; 2, low; 3, moderate; 4, severe. Ductal hyperplasia was scored according to its presence as 1, absent; 2, present. Acinar fibrosis was scored as 0, absent; 1, perivenular fibrosis with few septa and thin; 2, poor, thin septa with fibrous incomplete bridges between regions; 3, thin septa with extensive bridges between regions; 4, septa thickened with numerous bridges and nodular appearance. Peri-insular fibrosis was scored as 0, absent; 1, focal and mild increase of peripheral collagen deposition; 2, slight increase but diffuse; 3, moderate increase; 4, severe increase. Iron deposition was scored as 0, absent; 1, splashed positive macrophages; 2, mild positive macrophage accumulation; 3, moderate accumulations; 4, numerous Perls-positive macrophage accumulation.

### 3.3. Immunohistochemistry for Insulin and Glucagon Expression

β and α cells distribution in islets and their density (percentage of β and α cells per islet area) were assesed by using insulin and glucagon immunohistochemistry to indentify cell type. For all immunohistochemical techniques, pancreatic sections were dewaxed in xylene and rehydrated through graded alcohols to water. Antigen retrieval was performed with the PTLink module (Dako, Demark) using Dako low-pH Antigen Retrieval (AR) fluid (Dako, Demark) followed by several washes in water before being placed onto an AutostainerPlusLink (Dako, Demark) where the remainder of the immunohistochemical staining was performed using Envision™ FLEX (Dako, Demark). Briefly, sections were first placed in washing buffer followed by blockade of endogenous peroxidase with 3% hydrogen peroxide for 5 min. Then, the primary antibody Insulin (Clonal 2D11-H5) (Master Diagnóstica^©^ MAD-021340Q) and polyclonal Glucagon (Master Diagnóstica^©^ MAD-021325Q) were applied for 20 min, followed by another buffer wash and visualized with DAB for 10 min. Following a water wash, sections were counterstained in haematoxylin for 7 min, washed in water, dehydrated and coverslipped. For the image analysis, islets were defined as clusters of seven or more β cells in association with other morphologically identifiable endocrine cells. All data were obtained from immunostained sections. Islet image capture was performed using a DP72 camera Olympus (Tokyo, Japan) from an Olympus BX41 microscope (×20 objective) and analysed using *AnaliSYS Image* *Processing* software (Olympus). Islets were identified and images stored. Individual islet perimeters, islet size, total islet cell number, number and area of insulin positive (β) cells, (α) cells and the percentage of islet insulin and glucagon staining were semi-automatically determined. All insulin and glucagon positive cells in tissue sections were counted, including faintly positive cells.

### 3.4. Protein, Hormone and Metabolite Determinations

Frozen pancreas portions (around 100 mg each) were thawed and homogenized in 1 mL of alcohol:acid (50 parts of 95% ethanol:1 part of 10.2 N HCl) and then sonicated in a UP50H sonicator (Dr Hielscher, Teltow, Germany) for 3–5 pulses in ice bath. Homogenates were processed as was indicated by Duttaroy *et al.* [107]. Total insulin was measured by radio-immuno assay (RIA) by using the Coat-A-Count kit (Siemens, Los Angeles, CA, USA). Total glucagon was measured by RIA by using the Milipore kit (Billerica, MA, USA). Leptin was determined by ELISA (LINCO, St. Charles, MO, USA). The protein content of the pancreatic homogenates was determined by Bio-Rad assay.

Circulating glucose was determined by the glucose oxidase method coupled to the peroxidase reaction [108]. Circulating triglycerides were determined from coupled reactions of lipoprotein-lipase, glycerol-kinase, glycerol phosphate oxidase and peroxidase giving a colour end-adduct according to previously reported [109]. Circulating cholesterol and total lipids were determined as was indicated by Naito and David [110] and Bucolo and David [109] respectively. HOMA index was calculated according to Wallace and Matthews [111].

### 3.5. Statistics

Data are expressed as means ± standard error of the mean (SEM) of 6 animals. For quantitative parameters, prior to any statistical analysis, all variables were checked for normality and homogeneous variance using the Kolmogorov-Smirnoff and the Levene tests, respectively. When a variable was found not to follow normality, it was log-transformed and reanalyzed. To evaluate differences in the means between groups (virgin olive *vs.* sunflower and virgin olive *vs.* fish) one way ANOVA adjusted by Bonferroni correction was performed. For semiquantitative parameters (histological analysis), differences were analysed by the Kruskal-Wallis test, and a Mann-Whitney U test was used *a posteriori* to evaluate mean differences between groups. For all statistical analysis, a value of *p* < 0.05 was considered significant. Data were analysed using IBM^®^ SPSS^®^ Statistics 20 (IBM corp. Armonk, NY, USA) statistical package.

## 4. Conclusions

Despite that diets with a unique fat source over very long periods of time are not likely to occur in humans, animal models like the one presented here, could be very useful to examine possible effects of fat types predominant in a particular diet. In addition, these models could help to understand how diet can model organ histology in different physiopathological situations considering the difficulty to obtain certain human samples such as pancreas. Based on this approach, it was reported that different pancreatic compartments were affected according to the fat composition consumed, emphasizing the importance of dietary fatty acids in determining pancreatic structure. SO-rich diets mainly led to endocrine alterations with higher β-cell numbers and twice the insulin content, whereas diets rich in FO are associated to signs of pancreatic fibrosis of the elderly in rats [19]. Since there is a close relationship between mitochondria, oxidative stress and aging, here, we wanted to gain more insight into this process in pancreas by administering chronic low-dosages of CoQ. The main finding was that CoQ supplementation led to different results according to fat used in diet. It seems that in the context of endocrine pancreas structure and in particular β-cell, SO as the main fat in the diet, when it is supplemented on CoQ, resembles features associated to VOO, observing a reduction of cell mass but maintaining pancreatic insulin contents. However, results found in animals fed on FO could be interpreted in several ways. A first way might be that oxidative stress is not very important in relation to exocrine pancreas aging in the context of FO-rich diets. A second option might be that CoQ does not act as an antioxidant in this context. Nevertheless, these and other options will be investigated in future work. Therefore, CoQ may improve pancreas function when some deleterious dietary fats are being used as the basis of the diet. Thus, the effect of CoQ has to be assessed for each particular organ-compartment, taking into account the fat component of diet. The present study emphasizes from a histological point of view that chronic CoQ supplementation together with dietary fat type could play an important role in modulating aging in the pancreas, affecting different compartments of this organ in opposite ways.
